# Detection and Molecular Diversity of *Brucella melitensis* in Pastoral Livestock in North-Eastern Ethiopia

**DOI:** 10.3390/pathogens13121063

**Published:** 2024-12-03

**Authors:** Berhanu Sibhat, Haileeyesus Adamu, Kassahun Asmare, Johanna F. Lindahl, Ulf Magnusson, Tesfaye Sisay Tessema

**Affiliations:** 1 Institute of Biotechnology, Addis Ababa University, Addis Ababa P.O. Box 1176, Ethiopia; haileeyesus.adamu@aau.edu.et (H.A.); tesfaye.sisayt@aau.edu.et (T.S.T.); 2 College of Veterinary Medicine, Haramaya University, Dire Dawa P.O. Box 138, Ethiopia; 3 Division of Reproduction, Department of Clinical Sciences, Faculty of Veterinary Medicine and Animal Science, Swedish University of Agricultural Sciences, P.O. Box 7054, 750 07 Uppsala, Sweden; johanna.lindahl@slu.se (J.F.L.); ulf.magnusson@slu.se (U.M.); 4 Faculty of Veterinary Medicine, Hawassa University, Hawassa P.O. Box 05, Ethiopia; kassahun@hu.edu.et; 5 Department of Animal Health and Antibiotic Strategies, Swedish Veterinary Agency, 751 89 Uppsala, Sweden

**Keywords:** *Brucella melitensis*, brucellosis, goat, phylogenetic analysis, MLST, sheep, ST12, whole-genome SNP

## Abstract

Brucellosis is a neglected zoonotic disease affecting livestock and humans that remains endemic in Ethiopia. Despite its prevalence, only a few studies have identified *Brucella* species circulating in livestock in the country. This study aimed to determine the *Brucella* species responsible for infections in livestock in the Afar region of Ethiopia and characterize the isolates using whole-genome single nucleotide polymorphism (wgSNP) analysis and in silico multi-locus sequence typing (MLST). Comparisons were made between Ethiopian *Brucella* and regional and global isolates to determine their phylogenetic relationships. Surveys conducted in May and October–November 2022 in six villages of the Amibara district involved the collection of vaginal swabs (n = 231) and milk samples (n = 17) from 32 sheep and 199 goats kept by 143 pastoral households reporting recent abortions in the animals. *Brucella melitensis* was detected in three sheep and 32 goats, i.e., 15% (35/231) of animals across 20% (29/143) of households using bacterial culture and PCR-based methods (bcsp31, AMOS, and Bruce-ladder multiplex PCR). Of the 35 positive animals, *B. melitensis* was isolated from 24 swabs, while the remaining 11 were culture-negative and detected only by PCR. The genomic DNA of the 24 isolates was sequenced using Illumina Novaseq 6000 and assembled using the SPAdes pipeline. Nine- and 21-locus MLST identified 23 isolates as genotype ST12, while one isolate could not be typed. The wgSNP-based phylogenetic analysis revealed that the Ethiopian isolates clustered within the African clade and were closely related to isolates from Somalia. Several virulence factors responsible for adhesion, intracellular survival, and regulatory functions were detected in all isolates. No antimicrobial resistance genes associated with resistance to drugs commonly used for treating brucellosis were detected. Since *B. melitensis* is prevalent in sheep and goats, vaccination with the *B. melitensis* Rev-1 vaccine is the recommended strategy in these pastoral systems to protect animal and human health.

## 1. Introduction

Brucellosis is a neglected infectious disease impacting livestock and humans worldwide, leading to chronic conditions like undulant fever, weakness, myalgia, arthralgia, depression, anorexia, spondylitis, endocarditis, and meningoencephalitis [1,2,3]. A recent study estimated a global annual incidence of 2.1 million cases of human brucellosis, with Asia and Africa being the most affected regions, with 1.2–1.6 million and 0.5 million cases, respectively [4]. Humans contract the disease mainly through contact with infected animals, especially during livestock abortion, or by consuming unpasteurized dairy products [5,6]. Among the zoonotic *Brucella* species, *Brucella melitensis* is the most virulent, though *B. abortus*, *B. suis*, and *B. canis* can also infect humans [7].

Host-adapted *Brucella* species cause reproductive disorders in livestock, often affecting multiple hosts [8]. *Brucella abortus* leads to abortions, stillbirths, and reduced milk production, primarily in cattle, while *B. melitensis* causes similar problems primarily in small ruminants [9,10,11,12]. Camels are also susceptible to brucellosis and are infected by *B. melitensis* or *B. abortus,* depending on their proximity to infected small ruminants or cattle flocks/herds. Camels manifest similar clinical conditions to cattle and small ruminants, albeit to a milder degree [13].

In Ethiopia, brucellosis has historically been a neglected and non-notifiable disease. However, it has recently gained attention from public health and veterinary authorities, and it is now ranked as the country’s third most important zoonotic disease [14,15]. A national review of brucellosis research conducted over the past five decades indicates that the disease is endemic in livestock. These studies primarily employed serological tests, which do not differentiate specific *Brucella* species [16,17]. In this context, traditional mixed livestock farming practices—where different livestock species are raised by the same households, such as in the sedentary highland mixed crop–livestock systems and lowland pastoral livestock production systems [18,19]—pose an increased risk for cross-transmission of host-adapted *Brucella* spp. among various livestock species [20,21,22]. Consequently, knowing the specific *Brucella* species in an area is crucial for tracing the sources of infection and effectively targeting control measures. This underlines the necessity for *Brucella* species’ isolation, identification, and characterization [23].

Characterization of *Brucella* species using molecular methods, such as whole-genome-based diversity studies, multi-locus sequence typing (MLST), and multi-locus variable-number tandem repeat analysis (MLVA) in isolates from a specific geographic region, may play a crucial role in effectively targeting control measures. These methods also enhance understanding of transmission dynamics, aid in outbreak investigations and source tracing, and contribute to the overall knowledge of the global distribution of various *Brucella* species genotypes [24,25,26]. However, in the broader sub-Saharan Africa (SSA) region, there are only a few studies of this nature [27,28].

Some reports have identified *B. melitensis* from brucellosis cases in humans in Europe, traced back to sources in countries such as Somalia and Ethiopia [24,25,26]. However, detailed molecular studies on *B. melitensis* in livestock from sub-Saharan Africa remain scarce. To date, *B. abortus* has been isolated from samples collected from aborting dairy cows in the central Oromia region using bacteriological culture and biochemical methods and later confirmed through species-specific conventional PCR. Similarly, *B. melitensis* has been identified in goats slaughtered for meat [29], as well as in aborting goats from pastoral areas of Afar [30] and Borena [31] in Ethiopia. Further strain-level genotyping has been limited, with only one study providing a detailed analysis of *B. abortus* [28]. Therefore, this study aims to isolate and determine the molecular diversity of *Brucella* species through whole-genome single nucleotide polymorphism (wgSNP) and multi-locus sequence typing (MLST) analyses from the Afar region of Ethiopia.

## 2. Materials and Methods

### 2.1. Study Area

**Figure 1:**
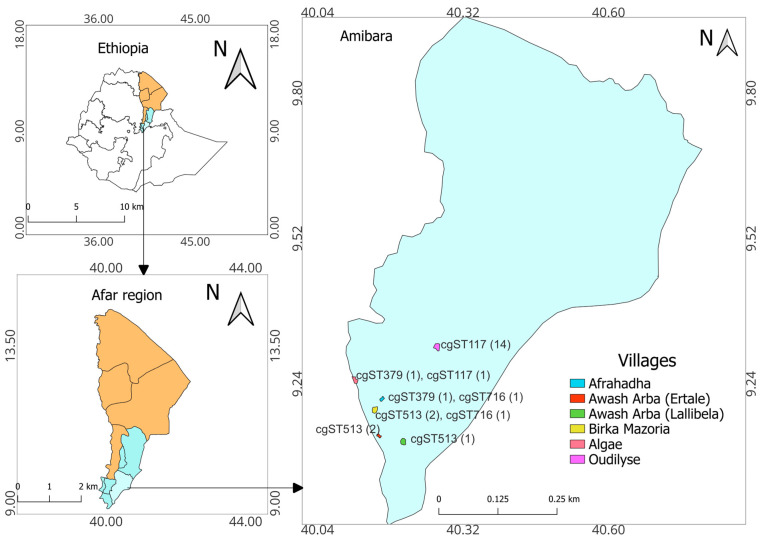
The study area. Core-genome MLST (cgMLST) profiles of B. *melitensis* isolates identified in this study are listed along with the study villages. The numbers in parentheses represent the number of isolates.

The study was conducted in the Amibara district in southern Afar Regional State (Figure 1). The district covers a total land area of 2941 km^2^ and is home to 63,000 inhabitants [32]. The maximum and minimum temperatures vary from 25 to 42 °C and 15.2 to 23.5 °C, respectively, and the average annual rainfall is 560 mm. The major rainy season extends from July to September, while the driest months are May and June (Melka-Worer Agrometeorological Station, 2008). Pastoralism is the major livelihood in the district. Livestock are kept primarily for milk and meat for self-sufficiency and sale [33].

### 2.2. Study Animals and Sampling

Based on reports of recent abortions in sheep, goats, and cattle, six villages in the Amibara district were selected for sample collection in collaboration with the district veterinary officer. Camels were excluded due to their absence in the villages during sampling periods. Two villages were visited in May 2022. Five villages, including one visited in May, were included in the October–November 2022 visit. The search for clinical abortion cases was conducted by going from house to house until every household (HH) in the villages was visited. Data collected from each HH included the reported history of abortions in livestock, including the season of abortions and any recent abortions within two weeks of the visit date.

In HHs with abortions, all animals that had aborted within two weeks at the time of sampling were included, and a questionnaire was completed to gather details on each of the affected animals, including species, parity, and stages of pregnancy. Whether the aborting animals were kept separately or with other herd/flock members was also recorded. Furthermore, body condition scoring (BCS) was conducted. A scale of 1–5 was used, where emaciated animals were assigned a value of ‘1’ and obese animals were assigned a value of ‘5’ [34,35]. Finally, vaginal swabs were collected using sterile cotton-tipped swabs and placed in sterile test tubes with Amies transport medium containing charcoal. When available, 20 mL of milk samples were collected from the same animals.

**Figure 2:**
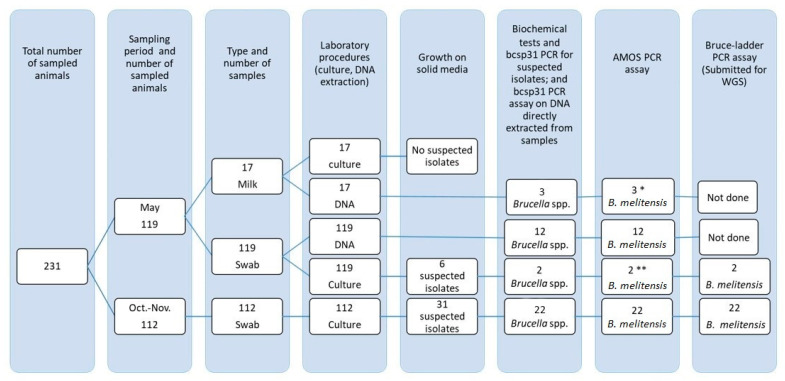
Sampling and sample processing for *Brucella* spp. identification from vaginal swabs and milk of sheep and goats. * Two of the animals with positive milk samples also had positive swabs. ** Both culture-positive animals also had PCR-positive swabs.

Overall, 248 samples (231 vaginal swabs and 17 milk) from 231 animals (199 goats and 32 sheep) were collected and processed for *Brucella* species identification as outlined in Figure 2. The samples were transported on ice to the district veterinary clinic within 3 h of collection and stored at 4 °C (swabs) and −20 °C (milk) until they were transported to Addis Ababa University, Institute of Biotechnology’s Health Biotechnology Laboratory (AAU-IoBL) and immediately processed upon arrival. The October–November samples were transported to AAU-IoBL within two days. Due to security incidents, the May samples were stored for an additional two weeks at the district veterinary clinic before being transported to AAU-IoBL.

### 2.3. Brucella Isolation and Characterization

The swab samples were each directly streaked onto two *Brucella* medium base (CM169) (Oxoid, UK) agar plates fortified with *Brucella* selective supplement (SR0083) (Oxoid, UK), and enriched with 5% horse serum. Milk samples were centrifuged at 4000× *g* for 15 min, and both the sediment and the top cream were spread over the surface of the *Brucella* medium base agar in duplicates. Subsequently, one plate underwent standard incubation at 37 °C in a conventional microbiological incubator. In contrast, the second plate was subjected to identical incubation conditions in an atmosphere containing 5–10% carbon dioxide. Presumptive *Brucella* colonies were confirmed using biochemical tests such as catalase, oxidase, urease, and various molecular assays [36].

### 2.4. Molecular Assays

#### 2.4.1. DNA Extraction

Three to five distinct *Brucella* colonies were selected from the surface of a four-day-old culture grown on the previously described selective *Brucella* medium. Total genomic DNA was extracted from the isolates using a commercial genomic DNA extraction kit, EZ-10 Spin Column Genomic DNA Minipreps Kit (BioBasic, Markham, ON, Canada), according to the protocol provided by the manufacturer. The genomic DNA was then stored at −20 °C until further use. DNA extraction from the swab and milk samples was conducted as described by Adamowicz et al. [37] and Romero [38], respectively.

#### 2.4.2. PCR Assays

Molecular assays, including a genus-specific *bcsp31* PCR assay [39] and a species-specific assay, AMOS multiplex PCR [40], were conducted on all swab and milk samples collected in May, as well as on all *Brucella* isolates identified on both visits. The Bruce-ladder PCR [41] was performed exclusively on culture-identified *Brucella* isolates. The genomic DNA samples from Bruce-ladder-confirmed *B. melitensis* isolates were used for whole-genome sequencing (WGS) (Figure 2).

### 2.5. Whole-Genome Sequencing, Assembly, and Bioinformatic Analyses

#### 2.5.1. DNA Library Preparation and Whole-Genome Sequencing

The genomic DNA samples were quantified using the Qubit dsDNA HS (High Sensitivity) Assay Kit (Life Technologies, Carlsbad, CA, USA), with readings taken on a Qubit 4.0 fluorometer (Life Technologies, Carlsbad, CA, USA). The quantity and purity of the DNA were assessed using a NanoDrop™ 1000 spectrophotometer (ThermoFisher Scientific, Wilmington, DE, USA). DNA libraries were prepared according to the manufacturer’s protocol using the TruSeq Nano DNA Library Preparation Kit (Illumina, San Diego, CA, USA). DNA sequencing was performed on the Illumina NovaSeq 6000 platform (Illumina, San Diego, CA, USA) at the Science for Life Laboratory (SciLifeLab) in Uppsala, Sweden.

#### 2.5.2. Retrieval of Regional/International Short-Reads from Public Databases

Eighty-four publicly available *B. melitensis* paired-end Illumina raw sequence reads and their associated metadata from Southern Europe, the Middle East, the Americas, North Africa, and SSA were downloaded from the National Center for Biotechnology Information (NCBI) sequence reads archive (SRA) and European Nucleotide Archive (ENA) using their accession numbers (last accessed 15 July 2024).

#### 2.5.3. Quality Assessment of Short Reads

All sequence reads from this study and those downloaded from public databases were subjected to quality assessment, including per base sequence quality, per base sequence content, per base GC content, per base N content, sequence length distribution, adapter content, etc. This was done using FastQC (Babraham Bioinformatics, Babraham Institute, Cambridge, UK (https://www.bioinformatics.babraham.ac.uk/projects/fastqc/ accessed on 16 July 2024).

#### 2.5.4. Genome Assembly

Following quality assessments, paired-end reads were de novo assembled using SPAdes version 3.15.5 [42] with the [–isolate] option using [--trusted contigs] along with the provision of the reference genome, *B. melitensis* biovar 1 strain 16M (GCF_000007125.1_ASM712v1). For one of the isolates, ETH2022-11, which could not be assembled using SPAdes, an IDBA de novo assembler version 1.1.3 [43] was employed. Contigs with a length of less than 500 bp were filtered out. Assemblies of reads downloaded from the public databases (NCBI and ENA) were conducted and processed as described for the isolates in this study.

#### 2.5.5. Genome Assembly Quality Assessment

The quality of genome assemblies was assessed using QUAST v5.0.2 [44] and BUSCO (Benchmarking Universal Single-Copy Orthologs) v5.5.0 [45] software packages. QUAST was used to evaluate genome quality metrics such as N50, N75, L50, L75, GC%, genome length, and number of contigs. BUSCO was used to assess the assembly completeness using the bacterial lineage-specific Orthodatabase v10 (bacteria_odb10), containing conserved orthologous bacterial genes. For *Brucella* spp. genomes, a total of 124 genes (BUSCOs) were searched in the mentioned database. Furthermore, to ensure that the assembled genomes were the expected *B. melitensis* organisms, GTDB-Tk v2.3.2 (Genome Taxonomy Database Toolkit) [46] was used to assign the taxonomic identity for the assembled genomes based on the Genome Taxonomy Database (GTDB) [47]. The commonly used average nucleotide identity (ANI) threshold target of 95% was used to consider an isolate to be *B. melitensis* [48]. The assembled genomes were annotated using Prokka [49].

#### 2.5.6. Pan-Genome Analysis

To investigate the pan-genome of the Ethiopian *B. melitensis* isolates, the Prokka-annotated genomes were put into Roary [50]. Roary classifies genes into different categories based on their presence across the isolates: core genes (99% to 100% presence), softcore genes (95% to less than 99% presence), shell genes (15% to less than 95% presence), and cloud genes (0% to less than 15% presence).

#### 2.5.7. Whole-Genome SNP (wgSNP) Calling and Phylogenetic Tree Construction

Variant SNP calling was conducted using the web-based CSIPhylogeny 1.4 pipeline available at the Center for Genomic Epidemiology, Technical University of Denmark (https://cge.food.dtu.dk/services/CSIPhylogeny/ accessed on 28 July 2024). The default parameters, 10× depth at SNP positions, a minimum of 10 bases distance between SNPs, a minimum Phred quality of 30 at each SNP position, and a minimum mapping quality of 25, were used. The web-based application allows only 100 genomes for SNP extraction. The reference genome *B. melitensis* bv1 st 16M (GCF_000007125.1_ASM712v1 was used as a reference for SNP calling [51]. The phylogeny derived from the SNPs was downloaded from the site as a Newick tree. Finally, the Interactive Tree of Life (iTOL) was used for phylogenetic tree annotation [52]. *B. abortus* 2308 (GCF_000054005.1_ASM5400v1) was used as an outgroup to root the phylogenetic tree.

#### 2.5.8. In Silico MLST Analyses

In silico MLST allelic profiles for the isolates were identified using a custom-written script and also by querying the assembled *B. melitensis* genomes against the *Brucella* MLST schemes, such as the 9-locus MLST [53], 21-locus MLST [54], core-genome MLST (cgMLST), and ribosomal MLST (rMLST) in the Public databases for molecular typing and microbial genome diversity (PubMLST) [55]. The diversity of the isolates in this study was analyzed by comparing them with global *B. melitensis* profiles stored in the PubMLST database. Nine-locus MLST profiles of global isolates used in developing the SNP tree in this study were also used as metadata for the global and regional wgSNP phylogenetic tree. GrapeTree [56] is used to visualize the minimum spanning trees of the 9- and 21-locus MLST profiles of the isolates in this study and those from the PubMLST database. In both scheme queries, the isolates were searched in the database by filling out the following phrases: “*Brucella* spp.” “=” “*B. melitensis*”; “country” “NOT contain” “Unknown”; Host species “NOT contain” “Unknown”, and “Not known”. The last search in the PubMLST database was on 20 August 2024.

#### 2.5.9. Predictions of Virulence Factors and Antimicrobial Resistance Genes

Virulence factors within the genomes of Ethiopian *B. melitensis* isolates were identified using Abricate v1.0.1 (https://github.com/tseemann/abricate, accessed on 2 August 2024). Abricate was employed to search for established virulence-associated genes by querying the National Center for Biotechnology Information (NCBI) and the Virulence Factor Database (VFDB). A web-based search for additional virulence factors was conducted using individual draft genomes of the *Brucella* isolates. Moreover, virulence factors that were not included in the databases but were described in the literature were in silico amplified from the draft genomes using primers detailed in the source literature via a web-based in silico PCR amplification tool (http://insilico.ehu.eus/user_seqs/PCR/amplify.php, accessed on 2 August 2024). Similarly, antimicrobial resistance-related genes were also queried using Abricate in Comprehensive Antibiotic Resistance Database (CARD), Antibiotic Resistance Gene-annotation (ARG-ANNOT), NCBI Bacterial Antimicrobial Resistance Reference Gene Database, and ResFinder databases (https://cge.food.dtu.dk/services/ResFinder/, accessed on 2 August 2024) [57].

## 3. Results

### 3.1. Abortions and Occurrence of Brucella melitensis

A total of 731 HHs were visited during the study, and abortions were reported in nearly one-fifth of them, as shown in Table 1. Households reported that abortions in livestock occurred throughout the year in the study area. Out of the 143 HHs that reported sheep and goat abortions at the time (within two weeks) of the visits, 90 HHs each reported a single abortion; 34 HHs had two, and 19 HHs had three to seven abortions. About 20% of the HHs with reported abortions had at least one animal that tested positive for *B. melitensis*. In all households (HHs) where abortions occurred, the affected animals were kept together with the rest of the flock /herd. Detailed clinical and animal-level data are presented in Supplementary Table S1.

### 3.2. Brucella *spp*. Detection and Isolation

Out of the 248 samples collected from 231 animals during both visits, *B. melitensis* was identified in 39 samples from 35 animals (15.1%) (four animals had two positive samples each) using culture, biochemical tests, and serial application of bcsp31, AMOS, and Bruce-ladder PCR assays (Figure 2). Thirty goats (15.1%) and five sheep (15.6%) were infected. *Brucella melitensis* was detected in the vaginal swabs as well as milk samples.

**Figure 3:**
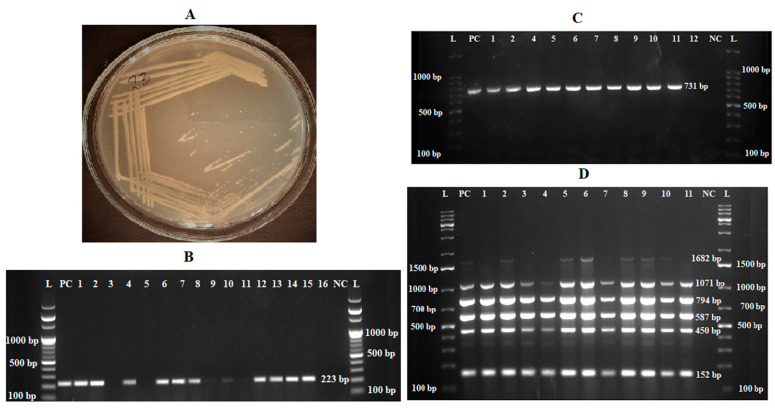
Characteristics of *Brucella melitensis* on culture media and gel images. (**A**) A four-day-old culture on *Brucella*-selective medium enriched with 5% horse serum. (**B**) bcsp31 PCR; L, 100 bp DNA molecular ladder; PC, positive control; 1, 2, 4, 6, 7, 8, 10, 12-15, *Brucella* spp.; 3, 5, 9, 11, and 16 negative samples; NC, negative control. (**C**) AMOS PCR; L, 100 bp DNA molecular ladder; PC, positive control; NC, negative control; No. 1–11, field isolates. (**D**) Bruce-ladder PCR; L, 1 kb-plus DNA molecular ladder; PC, positive control; NC, negative control; No. 1–11, field isolates.

Regarding bacteriological culture-identified isolates, of the 248 samples cultured on *Brucella* selective media, 37 had initial bacterial growth. All growths were observed from swab samples. Thirteen isolates were excluded based on a detailed examination of colonial morphology and biochemical tests. Typical colonies considered were pin-point, circular, honey-dew drop-like translucent colonies that were straw-colored as light passed directly from behind them and turned white to bluish-white when the plates were turned so that the light entered the colonies from an angle (Figure 3A). Twenty-four isolates tested positive for urease, catalase, and oxidase. These isolates had a 223 bp on bcsp31 *Brucella* genus-specific PCR (Figure 3B) and 731 bp amplicons of *B. melitensis* on species-specific AMOS PCR (Figure 3C). Similarly, Bruce-ladder multiplex PCR amplicon sizes characteristic of *B. melitensis*, namely, 1682 bp, 1071 bp, 794 bp, 587 bp, 450 bp, and 152 bp, were detected on gel electrophoresis using ethidium bromide (Figure 3D).

### 3.3. Genome Assembly

This study used the Illumina NovaSeq 6000 platform to sequence 24 *B. melitensis* isolates. The average number of reads generated was 20,943,420 (12,316,769 to 30,116,940), with an average read length of 151 bases and a GC content of 57%. The average size of the assembled draft genomes was 3,297,241.3 bp (range: 3,282,462 to 3,301,080) with an average of 12.75 contigs (range: 8 to 64), and an average GC content of 57.22% (range: 57.21% to 57.24%). The overall quality assessment of the assembled genomes is provided in Supplementary Table S2.

### 3.4. Pan-Genome Analysis

The pan-genome of the 24 *B. melitensis* isolates in this study constituted a total of 3215 genes, where 3084 were classified as core genes (95.9%) shared by ≥99% of the isolates, 15 (0.5%) as softcore genes present in ≥95% to <99% of the isolates, 55 (1.7%) as shell genes present in ≥15% to <95% of the isolates, and 61 (1.9%) as cloud genes present in <15% of the isolates.

### 3.5. Whole-Genome SNP (wgSNP) Analysis of Ethiopian Isolates

**Figure 4:**
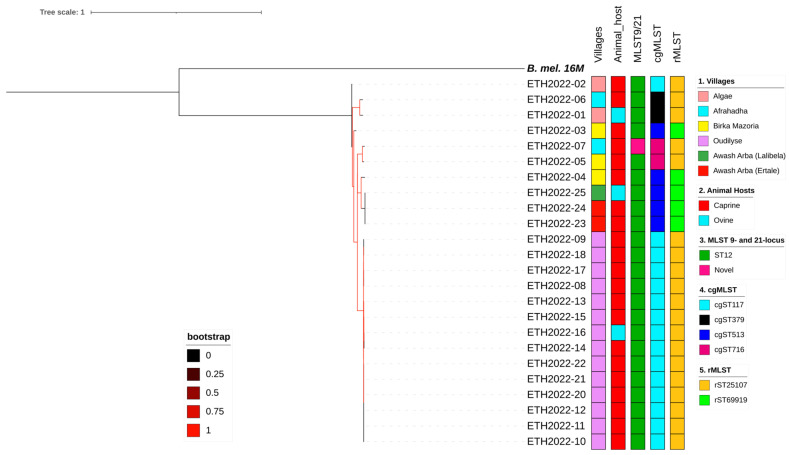
Whole-genome SNP-based maximum likelihood phylogenetic tree of the Ethiopian *B. melitensis*. The figure includes the metadata of the study villages, host species from which the *Brucella* were isolated, and the diversity of the isolates using the different multi-locus sequence type genotyping methods. Abbreviations: MLST9/21, multi-locus sequence typing using 9- and 21-locus schemes; cgMLST, core-genome multi-locus sequence typing; rMLST, ribosomal multi-locus sequence typing; ST, sequence type; B. mel. 16M, *B. melitensis* biovar 1 strain 16M reference genome.

Pairwise comparisons between the draft genomes of *B. melitensis* from Ethiopia revealed SNP differences ranging from 0 to 243 from each other and 1634 to 1665 from the reference genome. Three isolates obtained from two villages near Awash Arba town showed complete similarity, with no SNP differences (ETH2022-23, ETH2022-24, and Eth2022-25). Similarly, 14 isolates (ETH2022-08 to ETH2022-22) from one of the villages showed marked similarity, with only 0–7 SNP differences. Six isolates from the latter group (ETH2022-10, -11, -12, -20, -21, and -22) had no SNP differences among themselves. Isolates from other villages showed more diversity than the previously described villages (Figure 1 and Figure 4).

### 3.6. Regional and Global Comparisons

**Figure 5:**
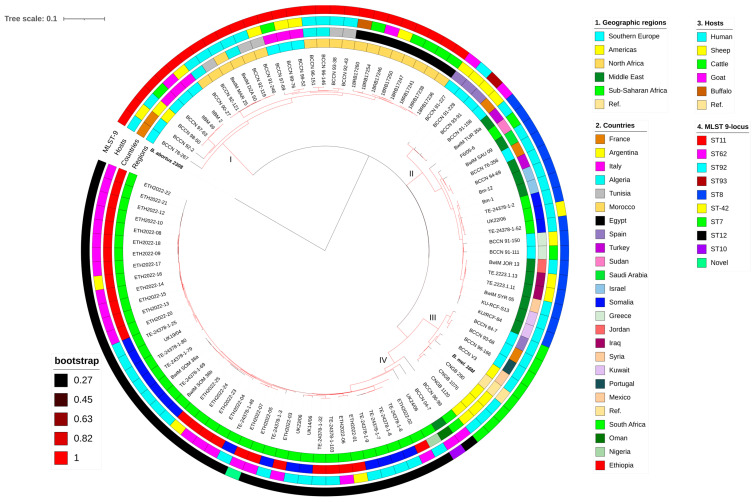
Whole-genome SNP-based maximum likelihood phylogenetic tree of Ethiopian *B. melitensis* from a regional perspective. The clades: I, Western Mediterranean; II, Eastern Mediterranean; III, American; IV, African. In the figure, the *B. melitensis* 16M reference genome is included for comparison, and the *B. abortus* 2308 reference genome is used as an outgroup for rooting the phylogenetic tree. All Ethiopian isolates in the current study are labeled ETH2022-xx. Isolates that start with TE-24378* and TE.2223* were originally named 2017-TE-24378* and 2019.TE.2223*, respectively (Supplementary Table S2).

All isolates in this study formed a large cluster with isolates from Ethiopia and Somalia and a few from other SSA countries from previous studies. All Ethiopian isolates (including those from other studies) are more closely related to Somali isolates than those from other African countries (Figure 5). Some isolates from Somalia and those in the current study exhibit small pairwise SNP differences ranging from 38 to 61, indicating a close genetic relationship. However, unlike some isolates from Somalia and Sudan (both neighboring Ethiopia) that cluster with the Eastern Mediterranean genotype common in southeastern and eastern Europe, the Middle East, and other regions of Asia, none of the Ethiopian isolates from this or previous studies cluster with other regional genotypes. Isolates from North African countries cluster with those from southern Europe, forming the Western Mediterranean clade. Notably, isolates from SSA, excluding those from Ethiopia and Somalia, are rare and were largely unavailable in public databases for most countries in the region (Figure 4 and Supplementary Figure S1).

#### In Silico MLST9, MLST21, cgMLST, and rMLST

All *B. melitensis* isolates in this study, except ETH2022-07, were identified as ST12 for 9- and 21-locus MLST schemes. ETH2022-07, however, had two unique alleles that were assigned *aroA* (32) and *omp25* (42) instead of the *aroA* (2) and *omp25* (10) commonly identified in all of the other 23 isolates in this study, and most of the SSA isolates. ETH2022-07 has a novel allelic profile and has not yet been assigned an ST genotype in the PubMLST database. There was more variability in cgMLST, where 15 cgST117, five cgST513, two cgST379, and two cgST716 were identified. Some villages had isolates with similar cgSTs, while others had mixed genotypes (Figure 1 and Figure 3). Only two rMLST types, rST25107 (19 isolates) and rST69919 (five isolates), were observed.

Regarding the global 9-locus MLST genotypes, an analysis of 778 isolates, including those from this study and other countries worldwide, revealed that the isolates in the current study cluster with the ST12 genotype commonly found in SSA. This genotype spans a broad geographical range, from Somalia in the east to Nigeria in the west, and Egypt in the north to South Africa in the south. In contrast, isolates from North African countries were predominantly classified as the ST11 genotype, which cluster together with isolates from southwestern European countries along the western Mediterranean Sea.

**Figure 6:**
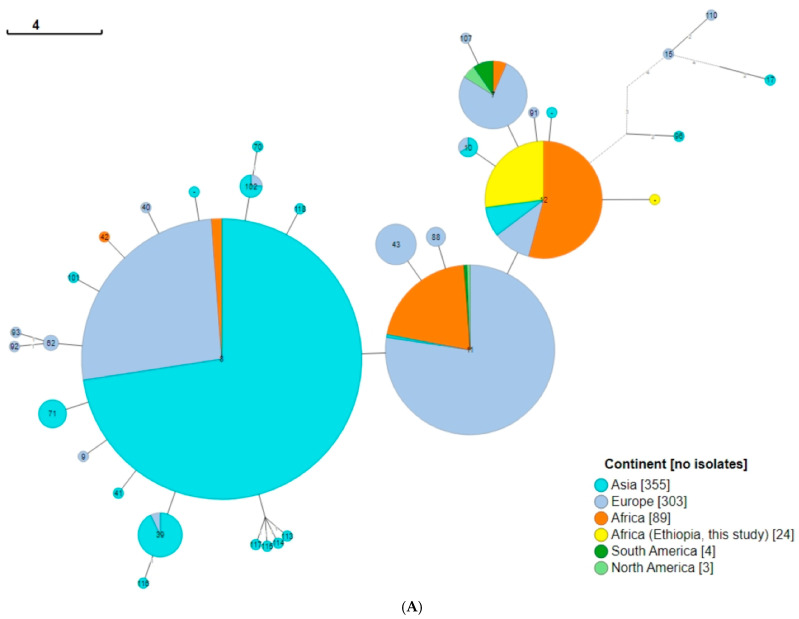

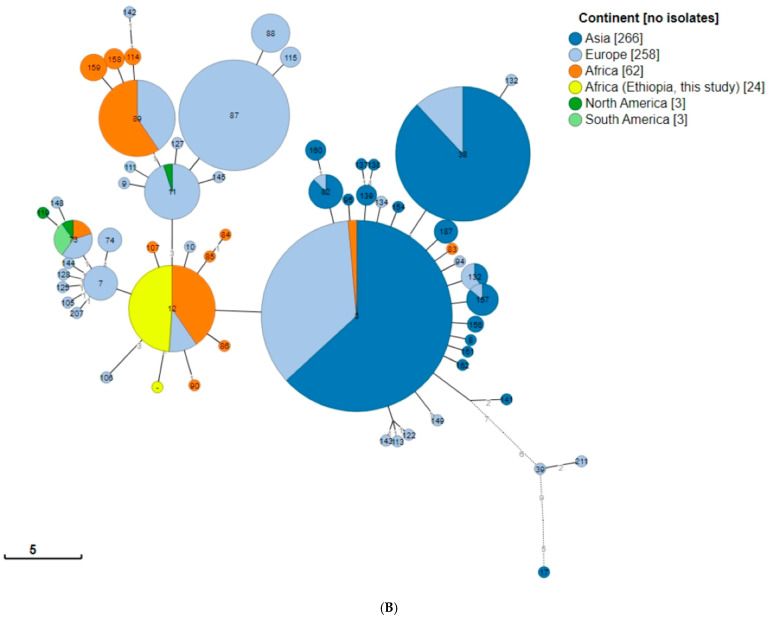
Minimum spanning tree: (**A**) 778 *B. melitensis* isolates, including 24 isolates from this study based on 9-locus MLST profiles derived in silico from assembled genomes. (**B**) Twenty-one-locus MLST profiles of 616 global isolates, including those in the current study. In this figure, numbers in the bubbles represent the sequence types, and those on the lines represent branch lengths.

Genotype ST12 seems rare in Egypt (n = 1/34), especially given the number of ST11 (n = 33/34) genotypes available in the PubMLST database (Supplementary Table S3). Sudan presents a unique case, with a few isolates in the PubMLST database classified as ST8, typical of the eastern Mediterranean, and ST7, common in the Americas. While Somalia is overwhelmingly represented by the African genotype (ST12), the PubMLST database also contains a few Somali isolates of ST42, which is closely related to ST8, as shown in Figure 6. Additionally, some Somali isolates are classified as ST8, as described in Figure 4 and Supplementary Figure S1.

Of the 85 individual isolates that form the ST12 node in Figure 6A, only 29 livestock isolates were represented, 23 (79.3%) being sheep and goats from the current study (Supplementary Figure S2C). Information on all the isolates and the metadata used in this figure are found in Supplementary Table S3.

Multi-locus sequence typing based on 21-locus analysis (Figure 6B) showed more diversity than the 9-locus MLST (Figure 6A). Of the four major 9-locus MLST profiles (ST7, ST8, ST11, and ST12), ST12 appears to be the least diverse, and its genotype diverged into only a few further genotypes following analysis of the 21-locus MLST. In both the 9- and the 21-locus schemes, Europe appears to have the most diverse isolates.

### 3.7. Virulence Factors and Antimicrobial Resistance

A custom-written script run on the VFDB for the Ethiopian *B. melitensis* isolates uncovered 43 virulence factors in all of the isolates, except one isolate (ETH2022-11) where one of the virulence factors, the cyclic beta-1,2-D-glucan synthetase (*cgs*) gene, could not be located. The identified genes include 31 genes associated with the biosynthesis of the lipopolysaccharides (LPS) of *Brucella* spp., the *virB* type IV secretion system (T4SS) and their effectors, and a gene responsible for intracellular survival (*cgs*). Additional web-based searches for virulence factors in the VFDB using individual draft genomes identified additional regulatory virulence factor genes, the *bvrR*/*bvrS* system, and several other genes involved in iron intake. In silico search for known published virulence genes using known sets of primers also uncovered several genes, including *mviN*, *omp25*, *omp31*, *znuA*, *bvfA*, *ure*, *vceC*, *betB*, *BPE275*, *BSPB*, *prpA*, *omp19*, and *perA*, in all of the isolates. A list of all the identified virulence factors and associated mechanisms is provided in Supplementary Table S4.

Regarding antimicrobial resistance (AMR) gene prediction in the genomes of the Ethiopian *B. melitensis*, no changes in genes associated with AMR were detected in NCBI, Argannot, and Resfinder databases. The CARD database, however, returned four genes in all of the isolates: *adeF* (efflux pump), *fosXCC* (antibiotic inactivation with fosfomycin thiol transferase), *Brucella suis mprF* (target alteration with defensin resistant *mprF*), and *qacG* (small multidrug resistance (SMR) antibiotic efflux pump responsible for resistance against disinfecting agents and antiseptics).

## 4. Discussion

This study found that abortions were common in small ruminants in the Afar pastoral region of Ethiopia, with almost 30% of the households having experienced recent abortions in May. Further, we could identify *Brucella melitensis* in 15% of sheep and goats with a recent history of abortion. This is similar to previous studies that successfully isolated *Brucella* from clinical samples obtained from small ruminants using bacteriological methods, later confirmed with conventional PCR, and identified only *B. melitensis* in goats from the Afar and Borena pastoral areas [29,30,31]. However, one previous study detected only *B. abortus*, in 35 of 36 small ruminants, but only species-specific conventional PCR from livestock serum samples was used [58]. Our study indicates that *B. melitensis* is one of the major etiological agents associated with abortions in sheep and goats in the area. No cattle abortions were observed during both visits, and no camels were available for sampling, as they had moved away from the villages in search of better browsing areas.

The identification of *B. melitensis* in nearly 20% of HHs with sheep and goat abortions in the study area is important for two reasons. First, it has significant public health implications. *Brucella melitensis* infects the mammary glands and is shed in the milk of the majority of infected goats following abortions and full-term deliveries during subsequent pregnancies [12,59]. In most HHs across pastoral regions of Ethiopia, milk is consumed raw, and in some areas, small ruminants, especially goats, provide milk for up to 95% of the population [60]. Additionally, studies have shown that public awareness of brucellosis in these communities is virtually non-existent. Pastoralists assist livestock deliveries bare-handed, and delivery or abortion products, such as fetal membranes and aborted fetuses, are discarded in surrounding fields without proper environmental or self-protection [60,61,62]. These practices create favorable conditions for transmitting *Brucella* to humans and animals.

Second, livestock in pastoral areas are herded together in communal pastures, where animals from multiple villages come in close contact [63]. The presence of *Brucella*-culture-positive animals in the herds, as observed in this study, is important for the transmission of brucellosis in livestock as these animals shed a large number of organisms in abortion materials and abortion fluids [12,64]. It is important to mention that not all *Brucella*-infected pregnant animals abort, while all infected pregnant animals shed the organism following delivery [22]. Evidence indicates that in livestock infected with *Brucella*, abortions predominantly occur during the first infected pregnancy. In contrast, subsequent pregnancies are typically carried to term despite ongoing infection and shedding of the organism [12,59]. Hence, the detection of *Brucella* infection based on the shedding of *Brucella* in the genital fluids of aborted animals alone most likely underestimates the true magnitude of the problem. Furthermore, the selective medium (Farrell’s medium) used for isolation of *Brucella* in the current study, while being the most commonly used, is known to inhibit the growth of some *Brucella* strains [65]. For these reasons, there is a need for public health authorities to raise awareness among pastoralists on the zoonotic transmission of brucellosis and its prevention in humans, and for veterinary authorities to implement control measures for the disease in livestock. Since sheep and goats are the primary reservoirs of *B. melitensis*, controlling the disease in these species is essential.

In this study, despite the limited geographic area covered during sampling, the various genotyping methods detected subtle genetic differences among the isolates. The wgSNP phylogenetic tree showed that four isolates, ETH2022-02, ETH2022-01, ETH2022-06, and ETH2022-03, were the most distant compared to the other isolates. Some villages at the farthest ends of the sampling area appeared to have the most similar isolates, while those in the middle had mixed genotypes. However, the genomic similarity observed in isolates from these villages might result from local outbreaks involving those specific strains at the time of the visit and, while interesting, may not exclude the existence of other strains as livestock from these villages move to each other’s pasture, especially during the dry season, based on the availability of pasture. Similarly, little diversity existed between the isolates when typed with MLST 9- and 21-locus schemes. Only one isolate, ETH2022-07, showed variation in two loci (*aroA* and *omp*25) common to both schemes. However, genotyping using cgMLST, which classifies isolates based on the presence of 1764 exact genes [66], demonstrated the existence of variations with the identification of four genotypes in the study area. Furthermore, the pan-genome analysis identified some non-core (shell and cloud) genes present only in a subset of the genomes, highlighting some genetic variation among the isolates. Therefore, typing methods with the highest resolution are required to differentiate between closely related strains circulating in the same geographic area.

The isolates from this study were closely related to 27 isolates previously reported from Ethiopia and Somalia. Except for two cattle isolates from Ethiopia, the sequences of which were made publicly available in late 2020, all isolates were obtained from human cases of brucellosis diagnosed in European countries, including Germany, Norway, Sweden, and the UK, with their sources traced back to Ethiopia and Somalia [24,25,26]. These European countries are known to be free from *B. melitensis* [67]. Notably, these studies traced *B. melitensis* isolates from human patients to their likely geographic origins, even though no *B. melitensis* genomes from livestock in Somalia or Ethiopia were available at the time in public databases such as GenBank or the European Nucleotide Archive.

This study provides direct evidence of the circulation of the bacteria in the pastoral livestock reservoir hosts from the region. This exemplifies how genotyping techniques such as WGS can enhance our understanding of the phylogeography of *B. melitensis*, offering valuable insights into tracing zoonotic disease outbreaks to their probable sources. The similarities between the isolates from Ethiopia and Somalia could be due to livestock movement across the national borders for trade (formal and informal) and in search of pasture and water, especially during severe drought [68,69].

The virulence of the *B. melitensis* isolates in this study is suggested by the fact that all isolates were recovered from clinical cases of abortions. This was also confirmed by the detection of virulence genes responsible for adherence, entry, intracellular trafficking, and multiplication within the target host cells. These genes include several genes involved in the biosynthesis of the LPS, intracellular survival and trafficking (*CβG*, *virB* T4SS), regulation (Two-component BvrR/BvrS system), and others. Similar genes were also reported in *B. melitensis* in Egypt [70] and Iran [71]. Additionally, genes previously described to be involved in the virulence mechanisms of *Brucella* spp., including *znuA*, *bvfA*, *ure*, *mviN*, *omp25*, *omp31*, [72], *vceC*, *betB*, *bpe275*, *bspB*, *prpA* [73], *omp19*, and *perA* [74] were identified in silico in all of the current isolates, implying that they are fully virulent.

Regarding antimicrobial resistance, no relevant genes were detected in three of the four databases searched. However, the CARD database identified *adeF*, *qacG*, *Brucella suis mprF*, and *fosXCC*. These genes correspond, respectively, to *bepE* (BME_RS08110), *emrE* (BME_RS05230)*, mprF* (BME_RS13545), and *fosX* (BME_RS13580) in the annotated genes of *B. melitensis* bv1 str 16M reference genome deposited in the NCBI’s Gene database. Interestingly, a similar study from Iran reported these genes in *Brucella* species, both with and without phenotypic antimicrobial resistance [71], and do not appear to contribute to resistance against the antimicrobial drugs typically used to treat human brucellosis.

As previously described, brucellosis is endemic in Ethiopia. However, no control program for the disease has been implemented in small ruminants in the region so far. This and other studies have identified isolated *B. melitensis* from cases of abortion in sheep and goats. These findings underscore the need to control the disease in livestock and reduce its zoonotic risk. Regardless of the biovar or genetic variant of *B. melitensis* circulating in small ruminants, and despite other prerequisites needed for brucellosis control and eradication, vaccination of sheep and goats remains the only practically viable strategy for managing the disease in low- and middle-income countries [75,76], particularly in extensive livestock production systems such as the pastoral systems examined here. Therefore, vaccination of sheep and goats with the live attenuated *B. melitensis* Rev-1 vaccine should be a central component of the control strategy in these species. One limitation of the current study is the small geographic area covered and the relatively short survey periods due to logistics and prevailing security conditions during the study. Future studies should include more extensive geographic coverage and longer sampling periods and consider seasons when livestock are accessible for sampling, especially in pastoral regions where livestock move from permanent settlement areas to dry-season grazing areas, which are often inaccessible by road.

## 5. Conclusions

*Brucella melitensis* is prevalent in sheep and goats in the Afar region of Ethiopia and is associated with abortion in a proportion of sheep and goats. Molecular analyses showed that the strains in this study are typical of the African clade clustered with those in the SSA and closely related to isolates from Somalia. Both 9- and 21-locus MLST schemes identified the isolates as ST12, except one with a novel profile. Small variations in isolates from the current study were better resolved with wgSNP and cgMLST analyses. There is a need for further isolation of *Brucella* spp. from cattle and camels in Afar to obtain the full picture of *Brucella* species circulating in livestock in the region. Therefore, brucellosis control in sheep and goats with the *B. melitensis* Rev-1 vaccine should be undertaken.

## Figures and Tables

**Table 1:** Abortions and diagnosed *B. melitensis* in the Amibara district of Afar, Ethiopia.

Month (2022)	No. Villages Visited ^§^	No. HHs Visited	No. HHs with Recent * Abortion (%)	No. HHs Positive for *B. melitensis* (%)	No. Animals Positive for *B. melitensis* (%)
May	2	312	89/312 (28.5)	13/89 (14.6)	13/119 (10.9)
Oct–Nov	5	419	54/419 (12.9)	17/54 (31.5)	22/112 (19.6)
Total	6	731	143/731 (19.6)	29/143 (20.3)	35/231 (15.2)

^§^ One of the villages was visited twice * within two weeks of the time of visits.

## Data Availability

The raw sequence reads associated with this study are available in the NCBI Sequence Read Archive (SRA) under BioProject ID PRJNA1179694, with BioSample numbers ranging from SAMN44501917 to SAMN44501940. The data presented in this study are available within the article or supplementary materials.

## Supplementary Materials

The following supporting information can be downloaded at: https://www.mdpi.com/article/10.3390/pathogens13121063/s1, Supplementary Table S1: Characteristics of sheep and goats from which *B. melitensis* was detected from vaginal swabs and milk samples of animals with a recent history of abortion during May, October, and November 2022; Supplementary Table S2: Genome quality assessment and pair-wise comparisons; Supplementary Table S3: MLST nine- and 21-locus schemes used for development of minimum spanning tree using GrapeTree; Supplementary Table S4: Virulence factors and mechanisms; Supplementary Figure S1: Phylogenetic relationship of Ethiopian *B. melitensis* with African isolates; Supplementary Figure S2: Nine and 21-locus MLST profiles of global *B. melitensis* by country and species.
