# Supplementary material for: Detection and Molecular Diversity of Brucella melitensis in Pastoral Livestock in North-Eastern Ethiopia
Source: Pathogens. 2024 Dec 3;13(12):1063. doi: 10.3390/pathogens13121063 (PMC11728775; doi:10.3390/pathogens13121063)
Supplement: Supplementary file 1 [file pathogens-13-01063-s001.zip › Supp. Fig. 1. Phylogenetic relationship of Ethiopian B. melitensis with African isolates.pdf]

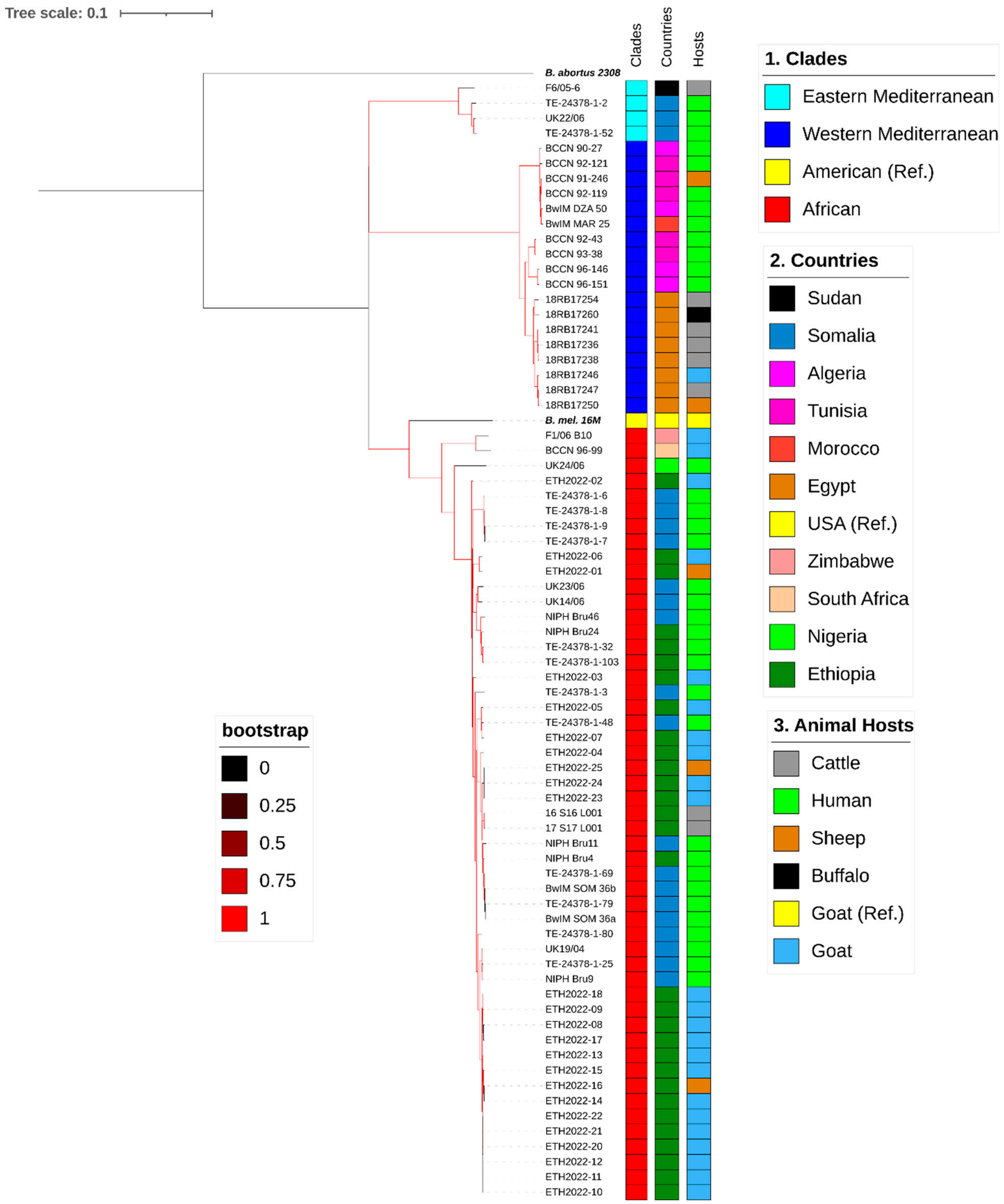

Supplementary Fig. 1. Whole-genome SNP based phylogenetic relationship of Ethiopian *B. melitensis* with isolates from other African countries. In this figure: B. mel. 16M, *B. melitensis* bvr1 16M reference genome (GCF_000007125.1) is used for comparison; B. abortus 2308 is a reference genome of *B. abortus* (GCF_000054005.1) used for rooting the phylogenetic tree as an outgroup. Ethiopian isolates in the current study are labeled as ETH2022-xx.
