# Supplementary material for: Detection and Molecular Diversity of Brucella melitensis in Pastoral Livestock in North-Eastern Ethiopia
Source: Pathogens. 2024 Dec 3;13(12):1063. doi: 10.3390/pathogens13121063 (PMC11728775; doi:10.3390/pathogens13121063)
Supplement: Supplementary file 1 [file pathogens-13-01063-s001.zip › Supp. Fig. 2. Nine and 21-locus MLST profiles of global B. melitensis by country and species.pdf]

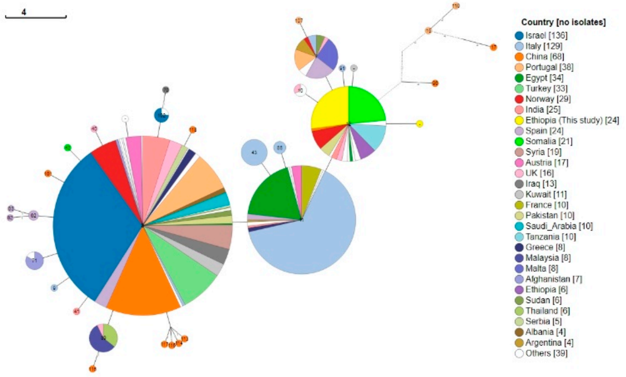

A. Nine-locus MLST profiles of *B. melitensis* from different countries of the world represented in the PubMLST database and the isolates from the current study.

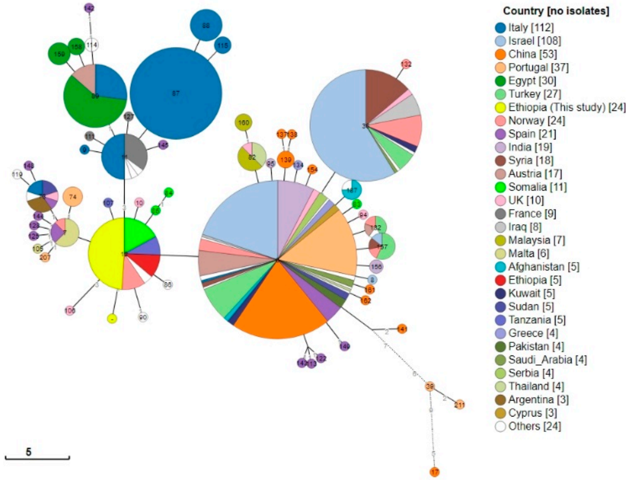

B. Twenty-one-locus MLST profiles of B. melitensis from different countries of the world represented in the PubMLST database and the isolates from the current study.

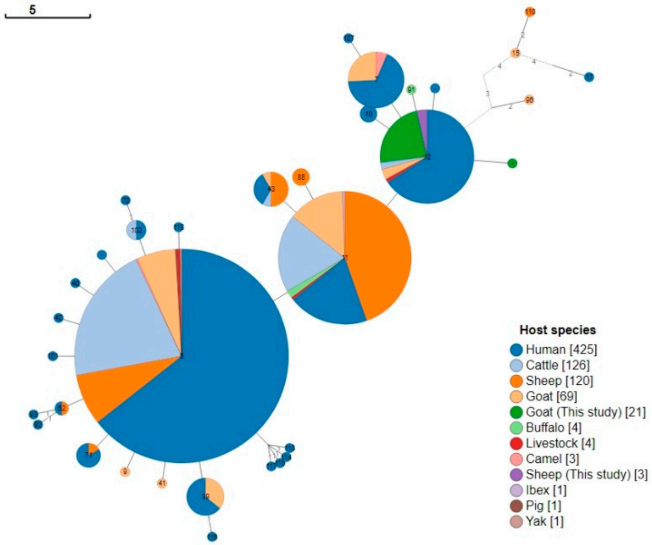

C. Nine-locus MLST profiles represented by host species from which *B. melitensis* was isolated. The figure represents isolates from countries of the world that had isolates deposited in the PuBMLST database. The majority of livestock isolates forming the ST12 node in this figure are from this study, no sheep isolates with ST12 were available in the PubMLST database at the time of access (Aug. 20, 2024).

Supplementary Fig. 2. Minimum spanning trees. Nine- and 21-locus MLST schemes of global *B. melitensis isolates* and those from this study analyzed and visualized by country (A., B.) and host species from which the bacteria were isolated (C.).
