# Supplementary material for: Detection and Molecular Diversity of Brucella melitensis in Pastoral Livestock in North-Eastern Ethiopia
Source: Pathogens. 2024 Dec 3;13(12):1063. doi: 10.3390/pathogens13121063 (PMC11728775; doi:10.3390/pathogens13121063)
Supplement: Supplementary file 1 [file pathogens-13-01063-s001.zip › Supp. Table 1. Characterstic of B. melitensis positive livestock.pdf]

Supplementary Table 1. Characteristics of sheep and goats from which *B. melitensis* was detected from vaginal swabs and milk samples of animals with a recent history of abortion during May, October, and November 2022.

Village	Sample No	Isolate No	Household No[§]	Month	Species	Parity	Abortion stage (months)	Body condition score	Culture result		bcsp31 PCR result		AMOS PCR result		Bruce-ladder PCR result (isolates)
									Vaginal swab	Milk	Vaginal swab/ isolate	Milk	Vaginal swab/ isolate	milk	
Algae	ETAF01	-	1	05/2022	Goat	3	4	2	-	NA	+ (s)	NA	+ (s)	NA	NA
	ETAF04	-	3	05/2022	Goat	1	4	2	-	-	+ (s)	+	+ (s)	+	NA
	ETAF05	-	3	05/2022	Sheep	3	4	2	-	NA	+ (s)	NA	+ (s)	NA	NA
	ETAF11	-	7	05/2022	Sheep	3	4	2	-	NA	+ (s)	NA	+ (s)	NA	NA
	ETAF18	ETH2022-01	12	05/2022	Sheep	2	3	2	+	NA	+ (s, i)	NA	+ (s, i)	NA	+
	ETAF23	ETH2022-02	16	05/2022	Goat	1	4	2	+	NA	+ (s, i)	NA	+ (s, i)	NA	+
	ETAF45	-	30	05/2022	Goat	2	3	2	-		+ (s)	NA	+ (s)	NA	NA
	ETAF54	-	36	05/2022	Goat	2	4	2	-	-	-	+	-	+	NA
	ETAF56	-	38	05/2022	Goat	1	3	2	-	-	+ (s)	+	+ (s)	+	NA
	ETAF105	-	77	05/2022	Goat	1	3	2	-	NA	+ (s)	NA	+ (s)	NA	NA
Oudilyse	ETAF37	-	37	05/2022	Goat	1	4	1	-	NA	+ (s)	NA	+ (s)	NA	NA
	ETAF82	-	82	05/2022	Goat	2	3	2	-	NA	+ (s)	NA	+ (s)	NA	NA
	ETAF87	-	87	05/2022	Goat	3	3	2	-	NA	+ (s)	NA	+ (s)	NA	NA
	ETAF176	ETH2022-08	111	11/2022	Goat	1	4	3	+	NA	+ (i)	NA	+ (i)	NA	+
	ETAF178	ETH2022-09	112	11/2022	Goat	1	3	3	+	NA	+ (i)	NA	+ (i)	NA	+
	ETAF179	ETH2022-10	113	11/2022	Goat	1	4	3	+	NA	+ (i)	NA	+ (i)	NA	+
	ETAF180	ETH2022-11	114	11/2022	Goat	2	4	4	+	NA	+ (i)	NA	+ (i)	NA	+
	ETAF181	ETH2022-12	115	11/2022	Goat	2	3	3	+	NA	+ (i)	NA	+ (i)	NA	+
	ETAF182	ETH2022-13	115	11/2022	Goat	1	3	3	+	NA	+ (i)	NA	+ (i)	NA	+

Village	Sample No	Isolate No	Household No[§]	Month	Species	Parity	Abortion stage (months)	Body condition score	Culture result		bcsp31 PCR result		AMOS PCR result		Bruce-ladder PCR result (isolates)
									Vaginal swab	Milk	Vaginal swab/ isolate	Milk	Vaginal swab/ isolate	milk	
	ETAF192	ETH2022-14	122	11/2022	Goat	1	3	3	+	NA	+ (i)	NA	+ (i)	NA	+
	ETAF193	ETH2022-15	122	11/2022	Goat	1	4	3	+	NA	+ (i)	NA	+ (i)	NA	+
	ETAF194	ETH2022-16	122	11/2022	Sheep	4	4	3	+	NA	+ (i)	NA	+ (i)	NA	+
	ETAF197	ETH2022-17	125	11/2022	Goat	1	4	3	+	NA	+ (i)	NA	+ (i)	NA	+
	ETAF201	ETH2022-18	125	11/2022	Goat	1	4	3	+	NA	+ (i)	NA	+ (i)	NA	+
	ETAF204	ETH2022-20	127	11/2022	Goat	1	4	3	+	NA	+ (i)	NA	+ (i)	NA	+
	ETAF207	ETH2022-21	128	11/2022	Goat	2	3	4	+	NA	+ (i)	NA	+ (i)	NA	+
	ETAF208	ETH2022-22	129	11/2022	Goat	2	4	3	+	NA	+ (i)	NA	+ (i)	NA	+
Afrahadha	ETAF152	ETH2022-06	104	10/2022	Goat	1	4	4	+	NA	+ (i)	NA	+ (i)	NA	+
	ETAF170	ETH2022-07	109	10/2022	Goat	2	4	3	+	NA	+ (i)	NA	+ (i)	NA	+
Birka Mazoria	ETAF124	ETH2022-03	91	10/2022	Goat	1	3	4	+	NA	+ (i)	NA	+ (i)	NA	+
	ETAF125	ETH2022-04	91	10/2022	Goat	1	3	4	+	NA	+ (i)	NA	+ (i)	NA	+
	ETAF131	ETH2022-05	95	10/2022	Goat	1	4	4	+	NA	+ (i)	NA	+ (i)	NA	+
Awash Arba (Ertale)	ETAF217	ETH2022-23	136	11/2022	Goat	1	4	4	+	NA	+ (i)	NA	+ (i)	NA	+
	ETAF221	ETH2022-24	138	11/2022	Goat	5	3	3	+	NA	+ (i)	NA	+ (i)	NA	+
Awash Arba (Lallibela)	ETAF227	ETH2022-25	143	11/2022	Sheep	1	4	3	+	NA	+ (i)	NA	+ (i)	NA	+

+, positive; -, negative; NA, not done or sample unavailable for sampling (in case of milk); i, PCR run on DNA from isolate; s, PCR run on DNA extracted from swab samples. [§] In all households, animals that had aborted were sampled while they were kept together with the herd/flock at the nighttime shelters.
