# Supplementary material for: Detection and Molecular Diversity of Brucella melitensis in Pastoral Livestock in North-Eastern Ethiopia
Source: Pathogens. 2024 Dec 3;13(12):1063. doi: 10.3390/pathogens13121063 (PMC11728775; doi:10.3390/pathogens13121063)
Supplement: Supplementary file 1 [file pathogens-13-01063-s001.zip › Supp. Table 4. Virulence factors and mechanisms.pdf]

Supplementary Table 4. Virulence-related genes identified in *Brucella melitensis* draft genomes from Ethiopia

A. Virulence-related genes identified through Abricate, gene annotation tools and, direct search using individual draft genomes in Virulence Factors Database (VFDB)

Virulence mechanisms	Components	Related genes
Adherence	Adhesins*	bmaC
		btaE, btaF
Immune modulation	LPS	*acpXL, fabZ, gmd, htrB, kdsA, kdsB, lpsA, lpsB/lpcC, lpxA, lpxB, lpxC, lpxD, lpxE, lpxK, manAoAg manBcore, manCcore, manCoAg, per, pgm, pmm, waaA/kdtA, wbdA, wbkA, wbkB, wbkC, wboA, wbpL, wbpZ, wzm, wzt*
	CβG (cyclic-β-1,2-glucan	*Cgs*§
	BtpA/Btp1/TcpB (Brucella Tir-domain containing protein A)	*btpA*
	BtpB (Brucella Tir-domain containing protein B)	*btpB*
Effector delivery system	virB type IV secretion system (T4SS)	*virB1, virB2, virB3, virB4, virB5, virB6, virB7, virB8, virB9, virB10, virB11, virB12*
	T4SS secreted effectors	ricA, vceC,
Nutritional/metabolic factors	Iron uptake – Brucebactin**	*dhbA, dhbB, dhbC, dhbE, entD, vibH/entF*
Regulation	Two-component BvrRS**	*bvrR, bvrS*

§identified in all genomes except ETH2022-11, *identified in all isolates through Prokka gene annotation tool, **identified in all isolates through a direct search in VFDB each draft genome,

B. Virulence factors associated with genes identified by *in silico* PCR using published sets of primers in all Ethiopian *B. melitensis* draft genomes in the current study.

Gene	Forward primer	Reverse primer	amplicon size	Reference
mviN	GCAGATCAACCTGCTCATCA	GCCATAGATCGCCAGAATA	344	[71] [73]
omp25	CGTACCTCACGGCTGGTATT	CGTACCGGCCAGATCATAGT	188	[71]
omp31	GCTGCTCCTGTTGACACCTT	GCTGAAATCGAACCCGTAAC	257	[71]
znuA	CTGGGTCCGAGCATGTTTAT	AGGCATCGAGTTTTTCTCCA	465	[71]
bvfA	CCCTTCGTCGATGTGCTGA	CCGCGCTGATTTCATCGCTG	1282	[71]
ure	GCTTGCCCTTGAATTCCTTTGTGG	ATCTGCGAATTTGCCGGACTCTAT	2214	[71] [73]
vceC	CGCAAGCTGGTTCTGATC	TGTGACGGGTAATTTGAAGC	482	[72]
betB	GCTCGAAACGCTGGATAC	AGGCGATGATTGACGAGC	393	[72]
bpe275	TGTCGCGGTCTATGTCTATC	AATGAGGACGGGCTTGAG	466	[72]
bspB	TATCCATGGTATATGCGCC	ATAAAGGCCGGGAATGAC	336	[72]
prpA	AACCTCAATGGATCGACC	ACGGTCGATAGCCTTGTC	672	[72]
omp19	TGATGGGAATTTCAAAAGCA	GTTTCCGGGTCAGATCAGC	550	[73]
perA	GGAACGGTGGCACTACATCT	GGCTCTCTGTGTTCCGAGTT	716	[73]
